# Alcohol-based hand rub consumption surveillance in German hospitals – latest results

**DOI:** 10.1186/2047-2994-4-S1-P293

**Published:** 2015-06-16

**Authors:** M Behnke, JO Clausmeyer, C Reichardt, P Gastmeier

**Affiliations:** 1 Institute of Hygiene and Environmental Medicine, Charité – University Medicine Berlin, Berlin, Germany

## Introduction

HAND-KISS is a unit-based surveillance system of alcohol-based hand rub consumption (AHC). It is part of the German national nosocomial infection surveillance system (KISS).

## Objectives

We analyzed the development in AHC between 2007 to 2014.

## Methods

Participating hospitals transfer data on patient days and AHC per unit once a year to HAND-KISS. The system calculates the data as AHC in milliliter (ml) per patient day (PD) stratified by unit type (intensive care units [ICU] and non-ICU) and specialty (medical, surgical, pediatrics, neonatal etc.). The distribution of all participating wards are published annually as HAND-KISS reference data.

To evaluate AHC changes over the years, we selected all hospitals and wards which continuously provided surveillance data over the whole period of 8 years. For all ICUs and non-ICUs we estimated the median AHC (interquartile range, IQR) for every year and compared the results.

## Results

188 hospitals with 334 ICUs and 1,954 non-ICUs transferred AHC data for 2007 (645 hospitals, 890 ICUs and 6,063 non-ICUs for 2014[g1]). 101 hospitals with 130 ICUs and 760 non-ICUs continuously provided surveillance data over a period of 8 years.

In 2007, the median AHC in the ICUs was 69 ml/PD (IQR, 52 ml/PD - 95 ml/PD), and in 2014 the result was 115 ml/PD (IQR, 84 ml/PD - 140 ml/PD) corresponding with an increase of 54%. The median AHC on non-ICUs was 15 ml/PD (IQR, 12 ml/PD - 18 ml/PD) in 2007 and 26 ml/PD (IQR 26 ml/PD - 32 ml/PD) in 2014 corresponding with an increase of 83%. The hospital-wide progression is 81%.

## Conclusion

HAND-KISS is widely accepted in Germany. It is an established benchmarking tool to characterize hand hygiene behavior in an individual hospital.

## Disclosure of interest

None declared.

